# Rapidly Progressive Dementia Presenting as Schizophrenia Spectrum Disorder: A Diagnostic Dilemma

**DOI:** 10.7759/cureus.58875

**Published:** 2024-04-23

**Authors:** Ishaan Gautam, Swapnil Aloney

**Affiliations:** 1 Psychiatry, Jawaharlal Nehru Medical College, Datta Meghe Institute of Higher Education and Research, Wardha, IND

**Keywords:** multidisciplinary approach, diagnostic challenges, immunomodulatory therapy, anti-nmda receptor antibodies, schizophrenia spectrum disorder, rapidly progressive dementia

## Abstract

Rapidly progressive dementia (RPD) presenting initially as schizophrenia spectrum disorder poses significant diagnostic challenges. We present the case of a 55-year-old woman initially diagnosed with schizophrenia spectrum disorder due to symptoms including social withdrawal, disorganized behavior, and psychosis. However, the rapid progression of cognitive decline and motor dysfunction prompted further investigation, leading to the diagnosis of anti-N-methyl-d-aspartate (NMDA) receptor antibody-mediated encephalitis. Cerebrospinal fluid analysis revealed the presence of anti-NMDA receptor antibodies, guiding targeted immunomodulatory therapy with intravenous immunoglobulin and corticosteroids. This resulted in significant clinical improvement, highlighting the importance of comprehensive diagnostic evaluation and timely initiation of immunomodulatory therapy in autoimmune-mediated RPD. This case underscores the complexities of overlapping psychiatric and neurological conditions and emphasizes the need for a multidisciplinary approach to diagnosis and management.

## Introduction

Rapidly progressive dementia (RPD) is characterized by a swift decline in cognitive and functional abilities over a short period, typically ranging from weeks to months. While RPD is most commonly associated with neurodegenerative disorders such as Alzheimer’s disease and frontotemporal dementia, a subset of cases may have autoimmune etiologies, including anti-N-methyl-D-aspartate (NMDA) receptor antibody-mediated encephalitis [1]. Anti-NMDA receptor antibody-mediated encephalitis is an autoimmune disorder characterized by the production of antibodies against the NMDA receptors in the brain. This condition predominantly affects young adults but can occur across all age groups. The clinical presentation often includes psychiatric symptoms such as psychosis, agitation, and hallucinations, followed by neurological manifestations, including seizures, movement disorders, and autonomic instability [2].

The diagnostic evaluation of RPD poses significant challenges, particularly when psychiatric symptoms precede cognitive decline. Patients may initially receive diagnoses of primary psychiatric disorders, delaying the identification of underlying neurological pathology. The timely recognition of autoimmune-mediated encephalitis is crucial, as prompt initiation of immunomodulatory therapy can improve outcomes [3]. Cerebrospinal fluid (CSF) analysis, neuroimaging studies (such as MRI), and autoimmune antibody panels play pivotal roles in the diagnostic workup of RPD. Detection of anti-NMDA receptor antibodies in CSF is diagnostic for anti-NMDA receptor antibody-mediated encephalitis and can guide targeted therapy [4].

Immunomodulatory treatments, including intravenous immunoglobulin (IVIg) and corticosteroids, have demonstrated efficacy in autoimmune-mediated encephalitis. However, these therapies’ optimal timing, duration, and sequencing remain areas of ongoing research [5]. This case report underscores the importance of considering autoimmune etiologies in the differential diagnosis of RPD, particularly in patients with overlapping psychiatric and neurological symptoms. The case also highlights the need for a comprehensive diagnostic evaluation and multidisciplinary approach to optimize patient care and outcomes.

## Case presentation

A 55-year-old woman from a rural background was admitted with primary complaints of diminished social interaction, irritability, low mood, and disorganized behavior. These symptoms later progressed to include increased forgetfulness, language impairment, and, ultimately, motor dysfunction, marked by a loss of motor control. Over time, she developed psychotic symptoms, including reduced social interaction, suspicion toward both family members and unfamiliar individuals, and increasingly disorganized behavior, such as involuntary passing of bodily waste, damaging household items, and burning clothing. Additionally, she exhibited inappropriate laughter spells and behavioral disturbances.

As the illness progressed, the patient experienced a shift from predominantly psychotic symptoms to symptoms indicative of dementia prodrome, including worsening forgetfulness, loss of manual dexterity, and slowed walking. These symptoms significantly impacted her daily life and socioeconomic status. The onset of symptoms was gradual and insidious, worsening in severity over three months in a step-ladder pattern. Previously, a homemaker engaged in occasional farm work, the patient had no apparent stressors or precipitating factors before reduced social interaction. This led to irritability and verbal altercations with family members, resulting in a diminished interest in household responsibilities. As the illness progressed, she experienced increased daytime sedation.

Behavioral disturbances, such as periods of isolation followed by episodes of inappropriate laughter and muttering, were noted early in the disease course. Suspicion and accusations of poisoning directed toward family members and visitors emerged later, alongside extensive disorganized behavior, depersonalization, and withdrawal from reality. Psychotic symptoms overlapped with features of RPD, characterized by escalating forgetfulness, irritability, language impairment, and motor coordination issues. Distinguishing the onset of rapid dementia was challenging due to the concurrent presence of psychotic symptoms. Dementia manifested as an inability to recall basic information, perform simple arithmetic, or recognize her surroundings, accompanied by a decline in motor function and withdrawal from verbal communication. The patient’s vital signs and blood pressure were within normal limits, and systemic examination revealed no notable findings. However, neurologic assessment indicated impairments in attention, fluency, working memory, recent memory, and visuospatial functions, while language abilities remained intact. Cranial nerve function and motor and sensory responses appeared normal, although frontal release signs and bilateral postural tremors were observed.

The clinical presentation suggested a possible diagnosis of sudden-onset, rapidly progressive major neurocognitive disorder, potentially associated with hypertension. Differentials considered included vasculitis, vascular dementia due to multi-infarct pathology, infectious etiologies such as prion diseases, and autoimmune causes. Routine laboratory investigations yielded normal results, including complete blood counts, liver and renal function tests, electrolyte levels, peripheral smear, thyroid function tests, and assessments for vitamin B12 and homocysteine. Glycated hemoglobin levels were within the normal range, and the erythrocyte sedimentation rate was mildly elevated at 10 mm in the first hour. Further tests for paraneoplastic antibodies, human immunodeficiency virus, and Venereal Disease Research Laboratory returned negative results. Normal findings were also noted on visual evoked potentials, echocardiography, and renal artery Doppler imaging, although unilateral nephrolithiasis was detected on abdominal ultrasonography. MRI of the brain revealed extensive white matter hyperintensities in the hippocampus, reduced left hippocampal volume, and minor hyperintensities in the frontal and temporal lobes. These findings were consistent with previous MRI results nine months prior, which showed similar fluid-attenuated inversion recovery and diffusion changes. CSF analysis revealed no cells, with elevated protein levels (75 mg/dL) and normal glucose levels (84 mg/dL).

Following the notable findings on MRI, a CSF tapping was performed to assess the autoimmune antibody panel, revealing the presence of anti-NMDA receptor antibodies, which provided a new perspective for diagnosis. Due to concerns regarding acute exacerbation of behavior and uncertainties surrounding the clinical significance of the positive antibodies, methylprednisolone pulsing was deemed inappropriate. Instead, the patient underwent a five-day course of IVIg therapy at a dosage of 0.4 mg/kg/day, administered from days 145 to 150 of combined psychiatric and medical inpatient admissions.

Over the following seven days, the patient’s mental state remained consistently labile, characterized by ongoing thought disorder and agitation. However, between days 9 and 12 post-IVIg treatment, there was a notable and sustained improvement in her mental state. By day 14 post-IVIg, she presented as lucid, with a reactive affect, exhibiting polite speech and behavior. Additionally, she demonstrated the capacity to comprehend medical discussions and recall conversations.

Despite subsequent diagnostic investigations, including CT of the chest, abdomen, and pelvis, positron emission tomography scan, MRI of the brain, and a second lumbar puncture performed on day 12 post-IVIg, no paraneoplastic source of NMDA receptor antigens, such as a teratoma, or any neurodegenerative disorders were identified. Subsequently, the patient underwent a five-day course of 500 mg daily intravenous methylprednisolone, as her improved mental state was deemed stable enough to undergo steroid therapy. This decision aligned with the increasingly recommended, more aggressive treatment approach in the medical literature. The treatment was well-tolerated, and she experienced progressive daily improvements in her mental state and cognition.

Upon discharge, she commenced rituximab therapy with a plan for continued administration every six months. At the outpatient review six months post-treatment initiation, she maintained her improved psychiatric state and was living independently in the community. She reported no hallucinations or delusions and denied any new neurological symptoms. Additionally, she noted improvements in her memory and was continued on the same psychiatric drug regimen. However, due to her stability, the psychiatric team began to taper her lorazepam gradually, and with ongoing stability expected with rituximab treatment, there may be potential to reduce her antipsychotics further. Given the uncertainty regarding the timing of the development of anti-NMDA receptor antibodies, ongoing management must account for the likelihood of an overlap syndrome involving underlying schizophrenia with superimposed anti-NMDA receptor antibody-mediated encephalitis. Both conditions are currently well-controlled with a combination of antipsychotic therapy and rituximab.

## Discussion

The presented case highlights the intricate interplay between psychiatric and neurological manifestations in RPD, underscoring the diagnostic challenges and therapeutic implications in such cases. The patient initially exhibited symptoms suggestive of schizophrenia spectrum disorder, including social withdrawal, disorganized behavior, and psychotic features. However, the subsequent rapid progression to cognitive decline and motor dysfunction prompted further investigation, ultimately leading to the diagnosis of anti-NMDA receptor antibody-mediated encephalitis.

Anti-NMDA receptor antibody-mediated encephalitis is a rare but increasingly recognized autoimmune disorder characterized by producing antibodies against the NR1 subunit of the NMDA receptor. These antibodies disrupt NMDA receptor function, leading to a wide range of neurological and psychiatric symptoms, including psychosis, cognitive impairment, seizures, and movement disorders [6]. The co-occurrence of psychiatric symptoms, such as hallucinations and delusions, often precedes the onset of neurological manifestations, posing a diagnostic challenge [7].

The diagnosis of anti-NMDA receptor antibody-mediated encephalitis in the presented case was facilitated by CSF analysis revealing the presence of anti-NMDA receptor antibodies. This underscores the importance of comprehensive diagnostic evaluation, including CSF analysis, in patients with overlapping psychiatric and neurological symptoms. Notably, CSF analysis has emerged as a valuable tool for diagnosing autoimmune-mediated encephalitis, with the detection of specific antibodies aiding in targeted therapy selection [8].

Managing anti-NMDA receptor antibody-mediated encephalitis typically involves immunomodulatory therapy to suppress the autoimmune response and restore NMDA receptor function. IVIg and corticosteroids are commonly used as first-line treatments, with plasma exchange and rituximab reserved for refractory cases [9]. In the presented case, IVIg therapy resulted in significant clinical improvement, highlighting the efficacy of immunomodulatory interventions in autoimmune-mediated RPD.

However, several challenges remain in managing anti-NMDA receptor antibody-mediated encephalitis, including treatment timing and duration, relapse risk, and long-term outcomes. While early initiation of immunomodulatory therapy is associated with better outcomes, the optimal treatment duration remains uncertain, with some patients requiring prolonged or maintenance therapy to prevent relapse [10]. Additionally, the long-term neurological sequelae of anti-NMDA receptor antibody-mediated encephalitis, including cognitive impairment and movement disorders, underscore the importance of long-term follow-up and rehabilitation [11].

## Conclusions

The presented case underscores the intricate nature of diagnosing and managing progressive dementia (RPD) rapidly when it presents with overlapping psychiatric and neurological symptoms, mimicking schizophrenia spectrum disorder. Through comprehensive evaluation and the identification of anti-NMDA receptor antibodies in CSF, the patient’s condition was ultimately attributed to autoimmune-mediated encephalitis. Timely initiation of immunomodulatory therapy, including IVIg and corticosteroids, led to significant clinical improvement, highlighting the importance of targeted interventions in autoimmune-mediated RPD. However, challenges persist in determining the optimal treatment duration and addressing long-term neurological sequelae. This case emphasizes the need for heightened awareness among clinicians regarding the diverse presentations of RPD and the role of autoimmune mechanisms in its pathogenesis, ultimately guiding more accurate diagnoses and improved patient outcomes.
